# Supplementary material for: Mental Health Support for Hospital Staff during the COVID-19 Pandemic: Characteristics of the Services and Feedback from the Providers
Source: Healthcare (Basel). 2022 Jul 18;10(7):1337. doi: 10.3390/healthcare10071337 (PMC9324679; doi:10.3390/healthcare10071337)
Supplement: Supplementary file 1 [file healthcare-10-01337-s001.zip › healthcare-1731994-supplementary.pdf]

Additional File S1: (.DOCX) Graphical representation of the study methodology.

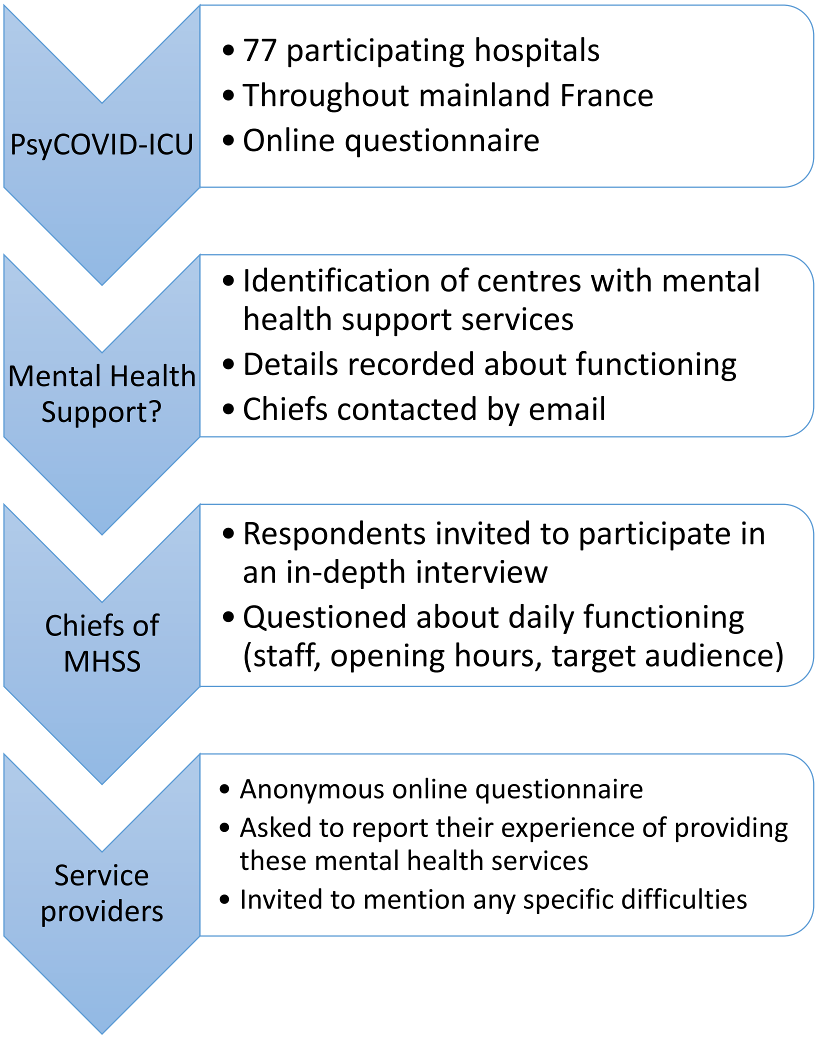

MHSS, mental health support services.

Additional File S2: (.DOCX) Study questionnaires regarding mental health support services during the epidemic.

1. **English translation of the questions relating to mental health support services from the initial PsyCOVID-ICU questionnaire**

1. Does your hospital have a mental health support service?

○ Yes ○ No

If **No**, is such a service…

- Planned : ○ Yes ○ No
- Needed: ○ Yes ○ No

If **Yes**, was this service…

- Set up specifically for the COVID-19 epidemic: ○ Yes ○ No
- Already in existence prior to the COVID-19 epidemic: ○ Yes ○ No

2. Please indicate the name and email address of the Chief of the Mental Health Support Service:

____

____

3. Do psychologists provide mental health support in this service?

○ Yes ○ No

If **Yes**, are they:

i. Psychologists from within our hospital: ○ Yes ○ No
ii. Psychologists from other hospitals: ○ Yes ○ No
iii. Private practice psychologists: ○ Yes ○ No

4. Do psychiatrists provide mental health support in this service?

○ Yes ○ No

If **Yes**, are they:

i. Psychiatrists from within our hospital: ○ Yes ○ No
ii. Psychiatrists from other hospitals: ○ Yes ○ No
iii. Private practice psychiatrists: ○ Yes ○ No

5. Please indicate the number of hours per day that this service is open: _______hours
6. How do potential users contact this service?
   a. Telephone helpline: ○ Yes ○ No

   b. Via a web-based interface: ○ Yes ○ No

      If **Yes**:

      - Free telephone call-back: ○ Yes ○ No
      - Make appointment online for a face-to-face meeting: ○ Yes ○ No

7. Can the mental health support service refer patients to other services?

   ○ Yes, to the occupational health unit
   ○ Yes, to the psychiatry unit
   ○ Yes, to other services
   ○ No

8. Do you have mental health support services for use by the families and/or relatives of patients?

○ Yes ○ No

9. For mental health support services targeting healthcare workers, which professions can use the service:

   ○ Staff from the intensive care unit
   ○ All healthcare professionals in the hospital
   ○ Staff from any hospitals or establishments that belong to our hospital group
   ○ Private practice physicians
   ○ Staff from nursing homes

10. Does the mental health support service work in collaboration with other organisations or services?

   ○ Yes ○ No
   If **Yes**, please give details: ___________________________________

2. Study Questionnaire for completion by providers of mental health support services

For Healthcare Workers staffing mental health support services:

- **Name of hospital** *(choice from a dropdown list) *:* ________________________________

- **Sex***: ○ Male ○ Female

- **Age***: ○ 20-34 years ○ 35-49 years ○ 50-65 years ○ More than 65 years

- **Profession** *(choice from a dropdown list)**: nurse/physician/psychologist/resident/medical student

- **Number of years' experience***:

○< 5 years ○ 5 to 10 years ○> 10 years

- Usual place of work: ○ Hospital ○ Private practice ○ Other. Please specify: _________

- If you work in a hospital, is it: ○ a university hospital ○ a specialist psychiatric hospital

- Do you have training in medico-psychological emergencies*: ○ Yes ○ No

- Is your usual place of work an occupational medicine department?*: ○ Yes ○ No

- If you are a psychologist, what type of initial training do you have? (choice from a dropdown list)

Clinical psychology/Psychanalysis/Social Psychology/Work Psychology/Developmental Psychology/Cognitive Psychology/Neuro-Psychology/Other (please specify)/Not applicable (I am not a psychologist)

- Is this the first time you have worked on a helpline providing mental health support for healthcare workers*?

○ Yes ○ No

If no:

- How many times have you done so previously:
- When was the last time (specify the date):

- When providing mental health support, I know where or to whom I can refer the patients: ○ Yes ○ No

- If appropriate, where did you refer callers after your contact with them via the helpline? (follow-up with our service, presence-based interview, medical consultation, follow-up in a medico-psychological care centre, hospitalisation, other (please specify).

- What was your comfort-level providing this service?*

On a scale from 0 to 10 (0 = I felt very uncomfortable; 10 = I felt totally comfortable)

- Do you think that additional training would be useful in providing mental health support services? ○ Yes ○ No

- If yes, what areas do you think the training should cover? *Free text answer*

- What difficulties did you encounter during your time providing mental health support during the epidemic? (specific situations, your perceptions, organisation…) *Free text answer*

- Would you provide this service again if asked? ○ Yes ○ No

- ⇴ If no, please tell us why not: ________________________

- Do you have any suggestions for improving the help offered by the mental health support service ○ Yes ○ No

⇴ If yes, please specify: ______________________________

Additional File S3: (DOCX) Details of the number of times each theme was mentioned by the respondents.

Theme	Number of occurrences (37 respondents)	
**By participating in the provision of mental health services, I had some difficult experiences**	**46 responses 49 themes**	
	N	%
Concerning the work organisation (practical/logistic organisation; uncertainty; hierarchy)	21	43
Concerning specific clinical situations (Non-COVID-related problems; refusal of care; low uptake)	12	24.5
Concerning my own emotional burden	11	22.4
Concerning recognition of my work on this service	3	6.1

Concerning my training, lack of experience in this activity	1	2.0
Concerning decision-making	1	2.0
**Need for additional training**	**21 responses**	**23 themes**
To improve my competence	18	78.3
Improve theoretical knowledge	11	47.8
Improve interview technique/telephone interviewing	7	30.4
To improve organisation of service delivery	5	17.4
**Improvements to this service are possible**	**13 responses**	**16 themes**
For the practical/logistic organisation	7	43.8
In the recognition given to this service and its providers	4	25
In the training for the professionals providing the service	3	18.7
In evaluating the efficacy of the service	2	12.5
